# Supplementary material for: ARTseq-FISH reveals position-dependent differences in gene expression of micropatterned mESCs
Source: Nat Commun. 2024 May 9;15:3918. doi: 10.1038/s41467-024-48107-5 (PMC11082235; doi:10.1038/s41467-024-48107-5)
Supplement: Supplementary file 3 — Description of Additional Supplementary Files [file 41467_2024_48107_MOESM3_ESM.pdf]

Description of Additional Supplementary Files:

Supplementary Data 1: Probe sequences in ARTseq-FISH. Probes and oligonucleotides used for detecting mRNAs and proteins in ARTseq-FISH.

Supplementary Data 2: Antibody list. The information of the antibodies used in this study, including names, cat numbers, suppliers, detecting total protein or non-phosphorylated protein.

Supplementary Data 3: Nanog smFISH probes. Probe sequences of the Nanog mRNA detection by smFISH.

Supplementary Data 4: Barcode. The barcode used to decode the targets after sequential hybridization. Each target is decoded by the readout colour in particular hybridization round.

Supplementary Data 5: Categories of targets. The categories of all the targets, and the targets used in the heatmaps in Figure 5.
