# Supplementary material for: ARTseq-FISH reveals position-dependent differences in gene expression of micropatterned mESCs
Source: Nat Commun. 2024 May 9;15:3918. doi: 10.1038/s41467-024-48107-5 (PMC11082235; doi:10.1038/s41467-024-48107-5)
Supplement: Supplementary file 9 — Reporting Summary [file 41467_2024_48107_MOESM9_ESM.pdf]

nature portfolio

Corresponding author(s): Maike M. K. Hansen
Wilhelm T.S. Huck

Last updated by author(s): Feb 27, 2024

Reporting Summary

Nature Portfolio wishes to improve the reproducibility of the work that we publish. This form provides structure for consistency and transparency in reporting. For further information on Nature Portfolio policies, see our Editorial Policies and the Editorial Policy Checklist.

Statistics

For all statistical analyses, confirm that the following items are present in the figure legend, table legend, main text, or Methods section.

n/a	Confirmed	
☐	☒	The exact sample size (*n*) for each experimental group/condition, given as a discrete number and unit of measurement
☐	☒	A statement on whether measurements were taken from distinct samples or whether the same sample was measured repeatedly
☒	☐	The statistical test(s) used AND whether they are one- or two-sided<br>*Only common tests should be described solely by name; describe more complex techniques in the Methods section.*
☒	☐	A description of all covariates tested
☒	☐	A description of any assumptions or corrections, such as tests of normality and adjustment for multiple comparisons
☐	☒	A full description of the statistical parameters including central tendency (e.g. means) or other basic estimates (e.g. regression coefficient) AND variation (e.g. standard deviation) or associated estimates of uncertainty (e.g. confidence intervals)
☒	☐	For null hypothesis testing, the test statistic (e.g. *F*, *t*, *r*) with confidence intervals, effect sizes, degrees of freedom and *P* value noted<br>*Give P values as exact values whenever suitable.*
☒	☐	For Bayesian analysis, information on the choice of priors and Markov chain Monte Carlo settings
☒	☐	For hierarchical and complex designs, identification of the appropriate level for tests and full reporting of outcomes
☐	☒	Estimates of effect sizes (e.g. Cohen's *d*, Pearson's *r*), indicating how they were calculated

Our web collection on statistics for biologists contains articles on many of the points above.

Software and code

Policy information about availability of computer code

Data collection	IQ3 and LAS X software was used for microscope image acquisition. BD FACSVerseTM was used to acquire flow cytometry data.
Data analysis	For the ARTseq-FISH data analysis, we cited the relevant python packages that were used in the custom designed software. A step by step guide on the operations of the software are described in the supplementary note 2. The code will be made publicly available before publication on github, including an archived version with DOI link, we will share the code with reviewers and editors. Image) was used to analyse representative images shown in the manuscript. FlowJo was used for flow cytometry data analysis.

For manuscripts utilizing custom algorithms or software that are central to the research but not yet described in published literature, software must be made available to editors and reviewers. We strongly encourage code deposition in a community repository (e.g. GitHub). See the Nature Portfolio guidelines for submitting code & software for further information.

Data

Policy information about availability of data

All manuscripts must include a data availability statement. This statement should provide the following information, where applicable:

- Accession codes, unique identifiers, or web links for publicly available datasets
- A description of any restrictions on data availability
- For clinical datasets or third party data, please ensure that the statement adheres to our policy

All data is available in the main text, the supplementary information, and supplementary tables. All other data supporting the findings of this study are available in a

publicly accessible repository.

Research involving human participants, their data, or biological material

Policy information about studies with human participants or human data. See also policy information about sex, gender (identity/presentation), and sexual orientation and race, ethnicity and racism.

Reporting on sex and gender	N/A
Reporting on race, ethnicity, or other socially relevant groupings	N/A
Population characteristics	N/A
Recruitment	N/A
Ethics oversight	N/A

Note that full information on the approval of the study protocol must also be provided in the manuscript.

Field-specific reporting

Please select the one below that is the best fit for your research. If you are not sure, read the appropriate sections before making your selection.

[x] Life sciences [ ] Behavioural & social sciences [ ] Ecological, evolutionary & environmental sciences

For a reference copy of the document with all sections, see nature.com/documents/nr-reporting-summary-flat.pdf

Life sciences study design

All studies must disclose on these points even when the disclosure is negative.

Sample size	Three independent biological replicates for 0, 12 and 24 hours after LIF withdrawal.<br>Four independent biological replicates for 48 hours after LIF withdrawal.<br>Three replicates of 0, 12, 24, and 48 hours mentioned above are the same set of experiments.<br>Sample sizes are indicated in the legends of Figures.<br>Sample size of the optimization and validation of ARTseq-FISH technique is indicated in the captions of the Supplementary Figures.<br>All the sample size are chosen based on previous experience and similar research (Y. Takei et al. Nature 590, 344-350 (2021).).
Data exclusions	Choice of padlock probes: we designed five unique padlock probes of Sox2 mRNA and three unique padlock probes of Nanog mRNA corresponding to different region of their mRNA sequence. We picked the most efficient padlock probe for the final experiment, which results in the most spots per cell.<br>Data analysis:<br>One replicate of 48 hours sample was not include in the analysis of Figure 4c and 5d-f due to the incomplete data acquisition of DAPI.<br>One replicate of 0, 12 and 24 hours sample were not included in the analysis of Figure 5d-e due to overwritten and failed image acquisition of DAPI.<br>We excluded the optimization results of permeabilization due to no obvious differences between the different permeabilization methods.
Replication	Three independent biological replicates for 0, 12 and 24 hours after LIF withdrawal.<br>Four independent biological replicates for 48 hours after LIF withdrawal.<br>Three replicates of 0, 12, 24, and 48 hours mentioned above are the same set of experiments.<br>Reproducibility (bulk counts of per target) was shown in Supplementary Fig. 21.
Randomization	Sample preparation and imaging for the “optimisation and validation of ARTseqFISH " (Supplementary Fig. 1-20) were preformed by a total of 4 different researchers to avoid biased. Image analysis and imaging was always performed by 2 different researchers to avoid biased. The results of the optimization and validation of the ARTseq-FISH were obtained by randomly sampling the fields of view.
Blinding	Image acquisition was performed in a randomised fashion per sample (Figure 1-7). Per micropattern imaging in the x-y direction was always performed from edge to centre to edge to ensure reproducibility and avoid errors in image analysis, however directionality of the imaging was randomised (if the images were captured by 60x oil objectives). Imaging in the z direction was always performed from top to bottom to ensure reproducibility and avoid errors in image analysis.

Reporting for specific materials, systems and methods

We require information from authors about some types of materials, experimental systems and methods used in many studies. Here, indicate whether each material, system or method listed is relevant to your study. If you are not sure if a list item applies to your research, read the appropriate section before selecting a response.

Materials & experimental systems

n/a	Involved in the study
☐	☒ Antibodies
☐	☒ Eukaryotic cell lines
☒	☐ Palaeontology and archaeology
☒	☐ Animals and other organisms
☒	☐ Clinical data
☒	☐ Dual use research of concern
☒	☐ Plants

Methods

n/a	Involved in the study
☒	☐ ChIP-seq
☐	☒ Flow cytometry
☒	☐ MRI-based neuroimaging

Antibodies

Antibodies used

GATA-6, supplier R&D; Catalog # MAB1700, Clone # 222228, Lot# JLM0219041
Brachyury, supplier R&D; Catalog # MAB20851-100, Clone # 1161B, Lot# CJDE0119071
Phospho-STAT3 (S727), supplier R&D; Catalog # MAB4934, Clone # 788335, Lot# CGVC0120111
Phospho-Akt1 (T308), supplier R&D; Catalog # MAB7419, Clone # 658320, Lot# CFVP0219101
Axin2, supplier R&D; Catalog # MAB6078, Clone # 354214, Lot# CCIT0319041
TCF3/E2A, supplier R&D; Catalog # MAB7650, Clone # 826927, Lot# CHKY0119051
CDX2, supplier Abcam; Catalog # ab220799, Clone # EPR2764Y, Lot# GR3345638-1
phospho-YAP1 (S127), supplier Abcam; Catalog # ab172374, Clone # EP1675Y, Lot# GR3271100-2
CDK4, supplier Sigma; Catalog # SAB1403657-1000G, Clone # 2G7, Lot# F9151-2G7
Rb, supplier Sigma; Catalog # ZRB1014-4X25UL, Clone # 2M15, Lot# Q3434660
STAT3, supplier Sigma; Catalog # ZRB1004-4X2, Clone # 4N11, Lot# 3248803
TCF7 (TCF1), supplier Sigma; Catalog # WH0006932M1-1000G, Clone # 1D2, Lot# H4242-1D2
Nanog, supplier Sigma; Catalog # ZRB1566-4X25UL, Clone # 4C17, Lot# Q3480264
p16-INK4a, supplier Sigma; Catalog # ZRB1437-4X25UL, Clone # 5F22, Lot# Q3394827
YAP, supplier Sigma; Catalog # MABS2029-1000G, Clone # 8G5, Lot# GR3271100-2
SMAD1, supplier Merck; Catalog # 05-1459, Clone # AS22, Lot# 3085594
AKT1, supplier Thermo Fisher Scientific; Catalog # CF504235, Clone # OTI4C11, CF504235
OCT4 (3H8L1.12), supplier Thermo Fisher Scientific; Catalog # 703927, Clone # 3H8L1.12, Lot# 2190822
CDK2c(1A6), supplier Thermo Fisher Scientific; Catalog # MA5-17052, Clone # 1A6, Lot# WA3165422
FOXA2 (9H5L7), supplier Thermo Fisher Scientific; Catalog # 701698, Clone # 9H5L7, Lot# 2283285
SOX17 (6H42L1), supplier Thermo Fisher Scientific; Catalog # 703063, Clone # 6H42L1, Lot# 2138114
Phospho-RNA pol II CTD (Ser5)(4H8), supplier Thermo Fisher Scientific; Catalog # MA1-46093, Clone # 4H8, Lot# UC278378
beta Catenin (CAT-5H10), supplier Thermo Fisher Scientific; Catalog # 13-8400, Clone # CAT-5H10, Lot# VD298608
Phospho-beta Catenin (Thr41, Ser45)(23H16L13), supplier Thermo Fisher Scientific; Catalog # 703638, Clone # 23H16L13, Lot# 2040068
SMAD2 (31H15L4), supplier Thermo Fisher Scientific; Catalog # 700048, Clone # 31H15L4, Lot# 2254790
Phospho-SMAD2 (Ser245, Ser250)(2H24L4), supplier Thermo Fisher Scientific; Catalog # 701723, Clone # 2H24L4, Lot# 2261072
Cyclin E (HE12), supplier Thermo Fisher Scientific; Catalog # 32-1600, Clone # HE12, Lot# UE288642
Phospho-Rb (Ser807, Ser811)(13H27L9), supplier Thermo Fisher Scientific; Catalog # 702097, Clone # 13H27L9, Lot# 2171451
N-Cadherin (CD325)(8C11), supplier Thermo Fisher Scientific; Catalog # 14-3259-82, Clone # 8C11, Lot# 2073578
E-Cadherin (CD324)(DECMA-1), supplier Thermo Fisher Scientific; Catalog # 16-3249-85, Clone # DECMA-1, Lot# 2178230
p21 (GT1032), supplier Thermo Fisher Scientific; Catalog # MA5-31479, Clone # GT1032, Lot# WA3163089
p53 (X77), supplier Thermo Fisher Scientific; Catalog # MA1-12549, Clone # X77, Lot# VL3138591
Cyclin D1 (AM29), supplier Thermo Fisher Scientific; Catalog # 33-3500, Clone # AM29, Lot# VJ316029
SNAIL1 (20C8), supplier Thermo Fisher Scientific; Catalog # 14-9859-82, Clone # 20C8, Lot# 2218477
SRSF3, supplier Thermo Fisher Scientific; Catalog # 334200, Clone # 7B4 (7B4A12), Lot# UE281515
Sox2, supplier Invitrogen; Catalog # MA531455, Clone # GT1876, Lot# YL4155926C
phospho-Smad1 (Ser463, 465), supplier Invitrogen; Catalog # 700047, Clone # 31H14L11 Lot# SG255223
Ki-67 (D3B5) Rabbit mAb, supplier Cell Signaling, Catalog # 9129, Clone # D3B5, Lot# 6
Cleaved Caspase-3 (Asp175) (5A1E) Rabbit mAb, supplier Cell Signaling, Catalog # 94530, Clone # 5A1E, Lot# 1

Validation

GATA-6 R&D; MAB1700, Clone # 222228, GATA-6 was detected in immersion fixed HCT-116 human colorectal carcinoma cell line using Mouse Anti-Human GATA-6 Monoclonal Antibody (Catalog # MAB1700) at 10 µg/mL for 3 hours at room temperature.
Brachyury R&D; MAB20851-100, Clone # 1161B, Brachyury was detected in immersion fixed BG01V human embryonic stem cells differentiated into mesoderm using Rabbit Anti-Human Brachyury Monoclonal Antibody (Catalog # MAB20851) at a 10 µg/mL for 3 hours at room temperature.
Phospho-STAT3 (S727) R&D; MAB4934, Clone # 788335, Daudi human Burkitt's lymphoma cell line was unstimulated (open histogram) or treated with 500 U/mL rhIFN-alpha for 20 minutes (filled histogram) and stained with Mouse anti-Human Phospho-STAT3 (S727) Molyclonal Antibody (Catalog # MAB4934) followed by APC-conjugated Anti-Mouse IgG Secondary Antibody (Catalog # F0101B).
Phospho-Akt1 (T308) R&D; MAB7419, Clone # 658320, Akt1 phosphorylated at T308 was detected in immersion fixed CCD-1070Sk human foreskin fibroblast cell line stimulated with Recombinant Human PDGF-BB (Catalog # 220-BB) using Mouse Anti-Human Phospho-Akt1 (T308) Mono-clonal Antibody (Catalog # MAB7419) at 25 µg/mL for 3 hours at room temperature. Cells were stained

using the Northern-Lights™ 557-conjugated Anti-Mouse IgG Secondary Antibody (red; Catalog # NL007).
Axin2, supplier R&D; Catalog # MAB6078, Clone # 354214, Axin-2 was detected in immersion fixed SW480 human colorectal adenocarcinoma cell line using Mouse Anti-Human Axin-2 Monoclonal Antibody (Catalog # MAB6078) at 10 µg/mL for 3 hours at room temperature. Cells were stained using the NorthernLights™ 557-conjugated Anti-Mouse IgG Secondary Antibody (red; Catalog # NL007).
TCF3/E2A, supplier R&D; Catalog # MAB7650, Clone # 826927, TCF-3/E2A was detected in immersion fixed frozen sections of E9.5 mouse embryo using Rat Anti-Mouse TCF-3/E2A Monoclonal Antibody (Catalog # MAB7650) at 10 µg/mL overnight at 4 °C. Tissue was stained using the NorthernLights™ 557-conjugated Anti-Rat IgG Secondary Antibody (red; Catalog # NL013).
CDX2, supplier Abcam; Catalog # ab220799, Clone # EPR2764Y, Immunohistochemistry (Formalin/PFA-fixed paraffin-embedded sections) analysis of Rat colon tissue sections labeling CDX2 with Purified ab76541 at 1:1000 dilution (0.8 µg/ml). Heat mediated antigen retrieval was performed using ab93684 (Tris/EDTA buffer, pH 9.0). Goat Anti-Rabbit IgG H&L (HRP) ab97051
was used as the secondary antibody.
Phospho-YAP1 (S127), supplier Abcam; Catalog # ab172374, Clone # EP1675Y, Dot blot analysis of YAP1 (pS127) peptide (Lane 1) and YAP1 non-phospho peptide (Lane 2) labelling YAP1 (pS127) with ab76252 at a dilution of 1/1000. ab97051 (Peroxidase conjugated goat anti-rabbit IgG (H+L)) was used as the secondary antibody at a dilution of 1/100000.
CDK4, supplier Sigma; Catalog # SAB1403657-1000G, Clone # 2G7, Immunofluorescence of anti-CDK4 (Catalog # SAB1403657) was used to detect CDK4 on Hela cells. Antibody concentration was 10 µg/mL.
Rb Sigma; ZRB1014-4X25UL, Clone # 2M15, Immunocytochemistry Analysis: A 1:100 dilution from a representative lot detected Rb (Retinoblastoma associated protein) in HeLa, A431, HepG2 and NIH 3T3 cells.
STAT3, supplier Sigma; Catalog # ZRB1004-4X2, Clone 4N11, Immunocytochemistry Analysis: A 1:150 dilution from a representative lot detected Stat3 in HUVEC, HeLa, A431, and NIH 3T3 cells.
Nanog, supplier Sigma; Catalog # ZRB1566-4X25UL, Clone # 4C17, Immunocytochemistry Analysis: A 1:100 dilution from a representative lot detected Nanog in NTERA-2 cells.
p16-INK4a Sigma; ZRB1437-4X25UL, Clone # 5F22, Immunocytochemistry Analysis: A 1:100 dilution from a representative lot detected p16-INK4a in HeLa, A431, HUVEC, and NIH3T3 cells.
YAP, supplier Sigma; Catalog # MABS2029-1000G, Clone # 8G5, Anti-YAP, clone 8G5, Cat. No. MABS2029, is a rat monoclonal antibody that detects Transcriptional coactivator YAP1 and has been tested or use in Immunocytochemistry, Immunofluorescence, Immunohistochemistry (Paraffin), Immunoprecipitation, and Western Blotting. Immunofluorescence Analysis: A representative lot detected YAP in Immunofluorescence applications (Miyamura, N.,et. al. (2017). Nat Commun. 8:16017).
SMAD1, supplier Merck; Catalog # 05-1459, Clone # AS22, Anti-SMAD1 Antibody, clone AS22 detects level of SMAD1 & has been published & validated for use in WB & IF. Immunocytochemistry Analysis: HeLa cells were fixed, permeabilized, and strained with Anti-SMAD1, clone AS22 (Cy3, red). Cells were co-stained with Phalloidin-AlexaFluorTM 488 (green, actin) and DAPI (blue, nuclear). Staining shows nuclear staining of SMAD1.
AKT1, supplier Thermo Fisher Scientific; Catalog # CF504235, Clone # OTI4C11, Anti-AKT1 mouse monoclonal antibody (TA504235) Immunofluorescent staining of COS7 cells transiently transfected by pCMV6-ENTRY AKT1(RC220257).
OCT4 (3H8L1.12), supplier Thermo Fisher Scientific; Catalog # 703927, Clone # 3H8L1.12, Knockdown of Oct-4 was achieved by transfecting iPSC cells with specific siRNA (Silencer® select Product # S10871; s10873). Immunofluorescence analysis was performed on iPSC cells (untransfected, panel a-d), transfected with Oct-4 specific siRNA (panel i-l) or non-specific scrambled siRNA (panels e-h). Cells were fixed, permeabilized, and labeled with Anti-Oct4 Recombinant Rabbit Monoclonal Antibody (Product # 703927, 1:16000 dilution), followed by Goat anti-Rabbit IgG (Heavy Chain) Superclonal™ Secondary Antibody, Alexa Fluor® 488 conjugate (Product # A27034, 1:2000).
CDK2c(1A6), supplier Thermo Fisher Scientific; Catalog # MA5-17052, Clone # 1A6. Immunofluorescence analysis of HeLa cells using CDK2 monoclonal antibody (Product # MA5-17052) (Green). Recommended dilution of this antibody is 1:200-1:1,000 for immunocytochemistry (ICC/IF).
FOXA2 (9H5L7), supplier Thermo Fisher Scientific; Catalog # 701698, Clone # 9H5L7, Immunofluorescence was performed on methanol fixed HepG2 cells for detection of FOXA2 using FOXA2 Recombinant Rabbit Monoclonal Antibody (Product # 701698, 2 µg/mL) and labeled with Goat anti-Rabbit IgG (Heavy Chain) Superclonal™ Secondary Antibody, Alexa Fluor® 488 conjugate (Product # A27034, 1:2000). For immunofluorescence analysis, iPSC differentiated to Definitive Endoderm were fixed and permeabilized for detection of endogenous FOXA2 using anti-FOXA2 Recombinant Rabbit monoclonal Antibody (Product # 701698, 1:100 dilution) and labeled with Goat anti-Rabbit IgG (Heavy Chain) Superclonal™ Secondary Antibody, Alexa Fluor® 488 conjugate (Product # A27034, 1:2,000). Panel a) shows undifferentiated iPSC cells, Panel b) is the image of iPSC differentiated to Definitive Endoderm showing nuclear localization of FOXA2.
SOX17 (6H42L1), supplier Thermo Fisher Scientific; Catalog # 703063, Clone # 6H42L1, For immunofluorescence analysis, iPS cells and iPS cells differentiated to definitive endoderm were fixed and permeabilized for detection of endogenous SOX17 using Anti-SOX17 Recombinant Rabbit Monoclonal Antibody (Product # 703063, 1:100) and labeled with Goat anti-Rabbit IgG (Heavy Chain) Superclonal™ Secondary Antibody, Alexa Fluor® 488 conjugate (Product # A27034, 1:2000). Nuclei (blue) were stained using ProLong™ Diamond Antifade Mountant with DAPI (Product # P36962) and cytoskeletal F-actin (red) staining using Rhodamine Phalloidin (Product # R415, 1:300) Panel a-d) shows representative IPS cells that were stained for detection and localization of SOX17 protein (green) with no signal compared to panel e-h) clearly demonstrating enhanced nuclear localisation of SOX17 in IPS cells differentiated to definitive endoderm.
Phospho-RNA pol II CTD (Ser5)(4H8), supplier Thermo Fisher Scientific; Catalog # MA1-46093, Clone # 4H8, Immunofluorescence analysis of NPhospho-RNA pol II CTD (Ser5) Monoclonal Antibody (4H8) was performed using 70% confluent log phase HCT 116 cells. The cells were fixed with 4% paraformaldehyde for 10 minutes, permeabilized with 0.1% Triton™ X-100 for 15 minutes, and blocked with 2% BSA for 45 minutes at room temperature. The cells were labeled with Phospho-RNA pol II CTD (Ser5) Monoclonal Antibody (4H8) (Product # MA1-46093) at 1:100 dilution in 0.1% BSA, incubated at 4 degree celsius overnight and then labeled with Donkey anti-Mouse IgG (H+L) Highly Cross-Adsorbed Secondary Antibody, Alexa Fluor Plus 488 (Product # A32766), (1:2000 dilution), for 45 minutes at room temperature (Panel a: Green). Nuclei (Panel b:Blue) were stained with ProLong™ Diamond Antifade Mountant with DAPI (Product # P36962). F-actin (Panel c: Red) was stained with Rhodamine Phalloidin (Product # R415, 1:300 dilution).
beta Catenin (CAT-5H10), supplier Thermo Fisher Scientific; Catalog # 13-8400, Clone # CAT-5H10, Immunofluorescent analysis of Beta-Catenin was done on 70% confluent log phase HeLa cells. The cells were fixed with 4% paraformaldehyde for 15 minutes; permeabilized with 0.25% Triton™ X-100 for 10 minutes followed by blocking with 5% BSA for 1 hour at room temperature. The cells were incubated with Beta-Catenin Mouse Monoclonal Antibody (Product # 13-8400) at 1.5 µg/mL in 1% BSA and incubated for 3 hours at room temperature and then labeled with Alexa Fluor 488 Rabbit Anti-Mouse IgG Secondary Antibody (Product # A-11059) at a dilution of 1:400 for 30 minutes at room temperature (Panel a: green). Journal of cellular physiology 2021 - 3 Figure TRPV4 affects

the nuclear localization of beta-catenin and represses beta-catenin/TCF-mediated transcription. (a) Immunofluorescence of endogenous TRPV4 channel (red) and beta-catenin (green) proteins in scattered MDCK cells in control conditions (DMSO) and after activation of the channel either with the addition of 4alpha-PDD (10 uM) for 30 min or by incubation in a hypotonic solution for 45 min. Lower row, cultures preincubated with RN-1734 (30 uM) 10 min before incubation in a hypotonic solution. (xz) Transversal sections at the level indicated with the discontinuous line. Arrows point to cells with nuclear localization of both TRPV4 and beta-catenin. Scale bars = 25 um.

Phospho-beta Catenin (Thr41, Ser45)(23H16L13), supplier Thermo Fisher Scientific; Catalog # 703638, Clone # 23H16L13, For immunofluorescence analysis, DU 145 cells were fixed and permeabilized for detection of endogenous Phospho-beta catenin (Thr41, Ser45) using Anti-Phospho-beta catenin (Thr41, Ser45) Recombinant Rabbit Monoclonal Antibody (Product # 703638, 1:100 dilution) and labeled with Goat anti-Rabbit IgG (Heavy Chain) Superclonal™ Secondary Antibody, Alexa Fluor® 488 conjugate (Product # A27034, 1:2000).

SMAD2 (31H15L4), supplier Thermo Fisher Scientific; Catalog # 700048, Clone # 31H15L4. Immunofluorescence analysis of SMAD2 was done on 70% confluent log phase TGF- beta treated HeLa cells (serum starved for 16 hours followed by treatment with 20 ng/mL TGF-beta for 1 hour). The cells were fixed with 4% paraformaldehyde for 15 minutes, permeabilized with 0.25% Triton X-100 for 10 minutes and blocked with 5% BSA for 1 hour at room temperature. The cells were labeled with SMAD2 Recombinant Rabbit Monoclonal Antibody (Product # 700048) at 1 µg/mL in 1% BSA and incubated for 3 hours at room temperature and then labeled with Alexa Fluor 488 Goat anti-Rabbit IgG Secondary Antibody (Product # A-11008) at a dilution of 1:400 for 30 minutes at room temperature (Panel a: green). Immunofluorescent analysis of SMAD2 in HeLa cells using a SMAD2 recombinant rabbit monoclonal antibody (Product # 700048) at a dilution of 2.5 µg/mL in the absence of peptide (left) or in the presence of the immunogenic peptide (right), followed by detection using an Alexa Fluor 488-conjugated goat anti-rabbit secondary antibody at a dilution of 1:1000.

Phospho-SMAD2 (Ser245, Ser250)(2H24L4), supplier Thermo Fisher Scientific; Catalog # 701723, Clone # 2H24L4, For immunofluorescence analysis NIH3T3 cells were fixed and permeabilized for detection of endogenous Smad2 [pS245+pS250] using Anti-Smad2 [pS245+pS250] Recombinant Rabbit Monoclonal Antibody (Product # 701723, 5 µg/mL) and labeled with Goat anti-Rabbit IgG (Heavy Chain) Superclonal™ Secondary Antibody, Alexa Fluor® 488 conjugate (Product # A27034, 1:2000). Immunofluorescence analysis of Smad2 pS245/pS250 using Anti-Smad2 pS245/pS250 Recombinant Rabbit Monoclonal Antibody (Product # 701723) shows induced expression of Smad2 pS245/pS250 in the nucleus of NIH/3T3 cells treated with TGF beta.

Phospho-Rb (Ser807, Ser811)(13H27L9), supplier Thermo Fisher Scientific; Catalog # 702097, Clone # 13H27L9, For immunofluorescence analysis, COLO 205 cells were fixed and permeabilized for detection of endogenous Phospho Rb (Ser807/ Ser811) using Anti- Phospho Rb (Ser807/Ser811) Recombinant Rabbit Monoclonal Antibody (Product # 702097, 2 µg/mL) and labeled with Goat anti-Rabbit IgG (Heavy Chain) Superclonal™ Secondary Antibody, Alexa Fluor® 488 conjugate (Product # A27034, 1:2000). Panel a) shows representative cells that were stained for detection and localization of Phospho-Rb (Ser807/Ser811) protein (green), Panel b) is stained for nuclei (blue) using SlowFade® Gold Antifade Mountant with DAPI (Product # S36938).

N-Cadherin (CD325)(8C11), supplier Thermo Fisher Scientific; Catalog # 14-3259-82, Clone # 8C11, This 8C11 antibody has been tested by flow cytometric analysis of HeLa cells. This can be used at less than or equal to 0.5 µg per test. A test is defined as the amount (µg) of antibody that will stain a cell sample in a final volume of 100 µL. Cell number should be determined empirically but can range from 10^5 to 10^8 cells/test. The 8C11 antibody has been tested by immunohistochemistry of formalin-fixed paraffin embedded tissue using low pH antigen retrieval at less than or equal to 10 µg/mL.

E-Cadherin (CD324)(DECMA-1), supplier Thermo Fisher Scientific; Catalog # 16-3249-85, Clone # DECMA-1. Immunofluorescence analysis of E-cadherin was performed using 90% confluent log phase MCF7 cells. The cells were fixed with 4% paraformaldehyde for 10 minutes, permeabilized with 0.1% Triton™ X-100 for 15 minutes, and blocked with 1% BSA for 1 hour at room temperature. The cells were labeled with CD324 (E-Cadherin) Monoclonal Antibody (DECMA-1) (Product # 16-3249-82) at 5 µg/mL in 0.1% BSA, incubated at 4 degree Celsius overnight and then labeled with Goat anti-Rat IgG (H+L) Superclonal™ Secondary Antibody, Alexa Fluor® 488 conjugate (Product # A-11006) at a dilution of 1:2000 for 45 minutes at room temperature (Panel a: green). International journal of molecular sciences 2021 - Figure 4 Tight junction proteins distribution in spheroids. A typical image of a spheroid. The presence of junction markers zona occludens-1 (ZO-1) and E-cadherin were confirmed via immunofluorescent staining: red, ZO-1; green, E-cadherin; blue, nuclei (DAPI).

p21 (GT1032), supplier Thermo Fisher Scientific; Catalog # MA5-31479, Clone # GT1032. p21 Monoclonal Antibody (GT1032) detects p21 Cip1 protein at nucleus by immunofluorescent analysis. Sample: MCF7 cells were fixed in 4% paraformaldehyde at RT for 15 min. Green: CDK4 protein stained by CDK4 antibody (Product # PA5-27827) diluted at 1:1,000. Immunocytochemistry-Immunofluorescence analysis of p21 was performed in Mock and treated HCT116 cells fixed in 4% paraformaldehyde at RT for 15 min. Green: p21 Monoclonal Antibody (GT1032) (Product # MA5-31479) diluted at 1:500.

p53 (X77), supplier Thermo Fisher Scientific; Catalog # MA1-12549, Clone # X77, Immunofluorescence analysis of Cellular tumor antigen p53 was performed using 70% confluent log phase MDA-MB-231 cells. The cells were fixed with 4% paraformaldehyde for 10 minutes, permeabilized with 0.1% Triton™ X-100 for 15 minutes, and blocked with 2% BSA for 45 minutes at room temperature. The cells were labeled with p53 Monoclonal Antibody (X77) (Product # MA1-12549) at 1:100 dilution in 0.1% BSA, incubated at 4 degree celsius overnight and then labeled with Donkey anti-Mouse IgG (H+L) Highly Cross-Adsorbed Secondary Antibody, Alexa Fluor Plus 488 (Product # A32766), (1:2000 dilution), for 45 minutes at room temperature (Panel a: Green). Antibody specificity was demonstrated by detection of differential basal expression of the target across cell models owing to their inherent genetic constitution. Immunofluorescence analysis using Anti-p53 Monoclonal Antibody (X77) (Product # MA1-12549), shows expression of p53 in MDA-MB-231 when compared to SK-OV-3.

Cyclin D1 (AM29), supplier Thermo Fisher Scientific; Catalog # 33-3500, Clone # AM29, Immunohistochemical staining of breast carcinoma tissue using Ms anti-Cyclin D1 (clone AM29) (Product # 33-3500). Molecular pathology: MP 2003 - Published figure using Cyclin D1 monoclonal antibody (Product # 33-3500) in Immunohistochemistry. Tested dilution of this antibody is 2-10 µg/mL.

SNAIL1 (20C8), supplier Thermo Fisher Scientific; Catalog # 14-9859-82, Clone # 20C8. Cell morphology was observed under microscope in ARPE-19 cells after stimulation of different doses of glucose. Magnification: x100. D VEGFA, Snail1, Occludin, E-cadherin, and Vimentin levels were examined by immunofluorescence in ARPE-19 cells in control or HG group (Cell death & disease 2021 - Fig. 1). Journal of Cancer 2020 - Figure 5 Knockdown of HOXC10 suppresses the WNT-EMT process in OSCC cell lines. C). FaDu cells and (D) SCC4 cells were treated with negative control (NC) and shHOXC10; representative immunofluorescence is shown, and fluorescence of N-Cadherin, Snail and E-cadherin was quantified; scale bar: 20 mum. The data are presented as the means +- SEM. **P<0.01 versus the control group.

SRSF3, supplier Thermo Fisher Scientific; Catalog # 334200, Clone # 7B4 (7B4A12). Antibody specificity was demonstrated by siRNA mediated knockdown of target protein. Hep G2 cells were transfected with SRSF3 siRNA and reduction of signal was observed in Western Blot using SRSF3 Monoclonal Antibody (Product # 33-4200). Knockdown validation info. Journal of virology 2016 - Published figure using SRSF3 monoclonal antibody (Product # 33-4200) in Western Blot and Immunohistochemistry.

Sox2, supplier Invitrogen; Catalog # MA531455, Clone # GT1876. SOX2 Monoclonal Antibody (GT1876) detects SOX2 protein by immunohistochemical analysis. Sample: Frozen sectioned adult mouse retina. Green: SOX2 protein stained by SOX2 Monoclonal Antibody (GT1876) (Product # MA5-31455) diluted at 1:250. Red: PKC alpha protein stained by PKC alpha antibody [GT1876] diluted at 1:250. Sox2 antibodies detects Sox2 proteins on embryonic mouse brain by immunohistochemical analysis. Sample: Frozen section of embryonic mouse brain (mE18.5). Green: GFAP antibody diluted at 1:500. Red: Sox2 antibody [GT1876] (Product # MA5-31455) diluted at 1:500. Immunohistochemistry analysis of SOX2 in frozen adult mouse retina using SOX2 monoclonal antibody (Product # MA5-31455) at a dilution of 1:250. Sample was then incubated with DAPI secondary antibody at a dilution of 1:250. SOX2 Monoclonal Antibody (GT1876) detects SOX2 protein expression at nucleus by immunohistochemical analysis. Sample: Frozen sectioned E13.5 Rat brain. Red: SOX2 protein stained by SOX2 Monoclonal Antibody (GT1876) (Product # MA5-31455) diluted at 1:250.

phospho-Smad1 (Ser463, 465), supplier Invitrogen; Catalog # 700047, Clone # 31H14L11. Immunofluorescence analysis of SMAD1/5 (pS463/465) was done on 70% confluent log phase HeLa cells. The cells were fixed with 4% paraformaldehyde for 15 minutes, permeabilized with 0.25% Triton X-100 for 10 minutes, and blocked with 5% BSA for 1 hour at room temperature. The cells were labeled with SMAD1/5 (pS463/465) Recombinant Rabbit Monoclonal Antibody (Product # 700047) at 2 µg/mL in 1% BSA and incubated for 3 hours at room temperature and then labeled with Alexa Fluor 488 Goat anti-Rabbit IgG Secondary Antibody (Product # A-11008) at a dilution of 1:400 for 30 minutes at room temperature (Panel a: green). Nuclei (Panel b: blue) were stained with SlowFade® Gold Antifade Mountant DAPI (Product # S36938). mmunofluorescent analysis of Phospho-SMAD1/5 pSer463/465 in HeLa cells using a Phospho-SMAD1/5 pSer463/465 recombinant rabbit monoclonal antibody (Product # 700047) at a dilution of 2.5 µg/mL in the absence of peptide (top left) and presence of phosphopeptide used as immunogen (top right) or non-phosphopeptide (bottom left), followed by detection using an Alexa Fluor 488-conjugated goat anti-rabbit secondary antibody at a dilution of 1:1000.

Ki-67 (D3B5) Rabbit mAb, supplier Cell Signaling, Catalog # 9129, Clone # D3B5. Confocal immunofluorescent analysis of the ventricular zone in P21 mouse brain using Ki-67 (D3B5) Rabbit mAb (green). Actin filaments were labeled with DyLight™ 554 phalloidin #13054 (red). Blue pseudocolor = DRAQ5® #4084 (fluorescent DNA dye). Confocal immunofluorescent analysis of HeLa cells using Ki-67 (D3B5) Rabbit mAb (green). Actin filaments were labeled with DY-554 phalloidin (red). Blue pseudocolor = DRAQ5® #4084 (fluorescent DNA dye).

Cleaved Caspase-3 (Asp175) (5A1E) Rabbit mAb supplier Cell Signaling, Catalog # 94530, Clone # 5A1E. Confocal immunofluorescent images of HT-29 cells, untreated (left) or Staurosporine #9953 treated (right), using Cleaved Caspase-3 (Asp175) (5A1E) Rabbit mAb (green), Alexa Fluor® 555 Phalloidin #8953 (red), and DRAQ5® #4084 (blue). Data were generated using the standard formulation of this product.

Before preforming complete ARTseqFISH protocol, all antibody-DNA conjugates were validated individually (see Supplementary Fig. 17).

Eukaryotic cell lines

Policy information about cell lines and Sex and Gender in Research

Cell line source(s)	Experiment were performed using mouse E14 embryonic stem cells, which were obtained from Hendrik Marks's group at Radboud University. Fucci mESCs were kindly provided by Menno Ter Huurne and Hendrik Marks at Radboud University. And the NANOG-GFP mESC line was kindly provided by Leor Weinberger's lab (Gladstone Institutes).
Authentication	All cell lines used in this study were authenticated via gene expression analysis and the morphology of mESCs.
Mycoplasma contamination	Cell lines were regularly tested for mycoplasma contamination and confirmed the absence of mycoplasma contamination.
Commonly misidentified lines (See ICLAC register)	No commonly identifies cell lines were used in this study.

Plants

Seed stocks	N/A
Novel plant genotypes	N/A
Authentication	N/A

Flow Cytometry

Plots

Confirm that:

- [x] The axis labels state the marker and fluorochrome used (e.g. CD4-FITC).
- [x] The axis scales are clearly visible. Include numbers along axes only for bottom left plot of group (a 'group' is an analysis of identical markers).
- [x] All plots are contour plots with outliers or pseudocolor plots.
- [x] A numerical value for number of cells or percentage (with statistics) is provided.

Methodology

Sample preparation	Flow cytometry analysis of cell cycle<br>mESCs were seeded in 1.9 cm2 wells (24-well format) with a density of 4.4x 103 cells/cm2. The wells were previously coated with 0.1% gelatin. Cells were seeded in serum/LIF medium and cultured for 24 hours. Then, cells were fixed in pre-chilled 70% ethanol for 30 minutes at -20°C and washed by lx PBS. Finally, cells were stained with 1 g/mL propidium iodide (Sigma-Aldrich, P4864-10ML) containing 10 g/mL RNaseA (VWR International B.V, 0675-250MG) for 15 minutes. After staining, cells were transferred to a flow cytometry tube and kept on ice until their immediate analysis in a BD FACS CaliburTM flow cytometer.<br>Flow cytometry analysis of Nanog-GFP mESC cell line in serum/LIF conditions.<br>The Nanog-GFP mESC line was kindly provided by Leor Weinberger's lab (Gladstone Institutes) 72. Nanog-GFP mESCs and non-labeled mESCs were seeded in 9.5 cm2 wells (6-well format) with a density of 4x 104 cells/cm2. The wells were previously coated with 0.1% gelatin. Cells were seeded in serum/LIF medium. 24 hours post-seeding, cells were detached with 400 L of 0.05% trypsin-EDTA for 2 minutes and resuspended in growing media. Cells were transferred to a flow cytometry tube and kept on ice until their immediate analysis in a BD FACS CaliburTM flow cytometer.<br>Flow cytometry analysis of CD24 expression in mESC<br>mESCs were seeded in 24-well plate with a density of 8400 cells/well. The wells were previously coated with 0.1% gelatin. Cells were seeded in serum/LIF medium and cultured for 96 hours, with a medium renewal at 48 hours of culture. Cells were stained with an anti-mouse CD24 antibody conjugated to alexa647 fluorophore (BioLegend, 101818) or an alexa647 isotype control antibody (BioLegend, 400626) for 30 minutes in serum/LIF media at 37 C. After staining, cells were washed with lx PBS and detached with accutase (StemCellTM technologies, 07922) for 5 minutes at 37 C, transferred to a flow cytometry tube and kept on ice until their analysis in a BD FACSVerseTM flow cytometer.
Instrument	BD FACS CaliburTM
Software	FlowJo software
Cell population abundance	No cell population were sorted in this study. We perform flow cytometry to identify the intensity distribution of selected markers.
Gating strategy	SSC vs. FSC gating to exclude debris. FSC-H vs. FSC-A gating to exclude doublets. For NANOG-GFP mESC, no GFP mESC sample was used to define the negative signals. For CD24 expression in mESC, alexa647 isotype control antibody was used to define the negative signals.

- [x] Tick this box to confirm that a figure exemplifying the gating strategy is provided in the Supplementary Information.
